# Position paper of the GMA Committee Interprofessional Education in the Health Professions – current status and outlook

**DOI:** 10.3205/zma001538

**Published:** 2022-04-14

**Authors:** Sylvia Kaap-Fröhlich, Gert Ulrich, Birgit Wershofen, Jonathan Ahles, Ronja Behrend, Marietta Handgraaf, Doreen Herinek, Anika Mitzkat, Heidi Oberhauser, Theresa Scherer, Andrea Schlicker, Christine Straub, Regina Waury Eichler, Bärbel Wesselborg, Matthias Witti, Marion Huber, Sebastin F. N. Bode

**Affiliations:** 1 Careum Foundation, Zurich, Switzerland; 2 Zurich University of Applied Sciences, Bachelor “Biomedical Laboratory Diagnostics”, Wädenswil, Switzerland; 3 Institute of Medical Education, University Hospital, LMU Munich, Munich, Germany; 4 Albert-Ludwigs-University Freiburg, Medical Faculty, Office of the Dean of Studies, Freiburg, Germany; 5 Charité – Universitätsmedizin Berlin, corporate member of Freie Universität Berlin and Humboldt-Universität zu Berlin, Deans Office of Study Affairs, Semester Coordination, Berlin, Germany; 6 University of Applied Sciences, Department of Applied Health Sciences, Division of Physiotherapy, Bochum, Germany; 7 Charité – Universitätsmedizin Berlin, corporate member of Freie Universität Berlin and Humboldt-Universität zu Berlin, Institute of Health and Nursing Science, Berlin, Germany; 8 Heidelberg University Hospital, Department of General Medicine and Health Services Research, Heidelberg, Germany; 9 fh gesundheit, fhg - Health University of Applied Sciences Tyrol, Innsbruck, Austria; 10 Bern University of Applied Sciences, Department of Health, Office for Interprofessional Teaching, Bern, Switzerland; 11 Witten/Herdecke University, Faculty of Health, Department of Human Medicine, Witten, Germany; 12 University of Freiburg, Faculty of Medicine, Department of General Pediatrics, Adolescent Medicine and Neonatology, Medical Centre, Teaching and Teaching Research/Teaching Development Working Group, Freiburg, Germany; 13 Protestant University of Applied Sciences Berlin, Bachelor of Nursing degree program, Berlin, Germany; 14 Fliedner Fachhochschule Düsseldorf, University of Applied Sciences, Nursing Education, Düsseldorf, Germany; 15 Zurich University of Applied Sciences, Department of Interprofessional Teaching and Practice, Winterthur, Switzerland; 16 Ulm University, Ulm University Medical Center, Department of Pediatrics and Adolescent Medicine, Ulm, Germany

**Keywords:** interprofessional learning, interprofessional education, interprofessional collaborative practice, competencies, evaluation, networks

## Abstract

In the wake of local initiatives and developmental funding programs, interprofessionality is now included in national curricula in the German-speaking countries. Based on the 3P model (presage, process, product), this position paper presents the development of interprofessional education in recent years in Germany, Austria and Switzerland and places it in an international context. Core aspects as legal frameworks, including amendments to occupational regulations as well as the formation of networks and faculty development are basic requirements for interprofessional education. New topics and educational settings take shape in the process of interprofessional education: patient perspectives and teaching formats, such as online courses, become more important or are newly established. The influence of the COVID-19 pandemic on interprofessional education is explored as well. Among many new interprofessional courses, particularly the implementation of interprofessional training wards in Germany and Switzerland are positive examples of successful interprofessional education. The objective of interprofessional education continues to be the acquisition of interprofessional competencies. The main focus is now centered on evaluating this educational format and testing for the corresponding competencies. In the future, more capacities will be required for interprofessional continuing education and post-graduate education. Structured research programs are essential to ascertain the effects of interprofessional education in the German-speaking countries.

In this position paper the GMA committee on interprofessional education encourages further advancement of this topic and expresses the aim to continue cooperating with other networks to strengthen and intensify interprofessional education and collaboration in healthcare.

## Abstract (de)

Das Thema der Interprofessionalität in den deutschsprachigen Ländern hat nach lokalen Initiativen und Förderprogrammen in nationale Ausbildungspläne Einzug erhalten. Angelehnt an das 3P Modell („Presage, Process, Product“; dt. in etwa: Rahmenbedingungen, Prozess, Produkt/Ergebnis) zeigt das nun neu vorliegende Positionspapier die Entwicklung interprofessioneller Ausbildung der letzten Jahre in Deutschland, in Österreich und in der Schweiz auf und setzt diese in einen internationalen Kontext. Neben rechtlichen Aspekten wie Veränderungen in den Berufsgesetzen zählen Netzwerkbildung und Fakultätsentwicklung zu den identifizierten Rahmenbedingungen. Im Prozess der interprofessionellen Ausbildung kristallisieren sich neue Themenfelder und Bildungssettings heraus, die Perspektive der Patient*innen und didaktische Formate wie Online-Angebote nehmen eine wichtigere Rolle ein bzw. wurden neu etabliert. In einem Exkurs werden darüber hinaus die Einflüsse der COVID-19-Pandemie auf interprofessionelle Ausbildung beleuchtet. Neben vielen neuen interprofessionellen Lernangeboten ist die Schaffung interprofessioneller Ausbildungsstationen in Deutschland und der Schweiz als Positivbeispiel für gelungene interprofessionelle Ausbildung hervorzuheben. Das Ziel interprofessioneller Ausbildung ist weiterhin der Erwerb interprofessioneller Kompetenzen. Im Fokus stehen dabei nun die Evaluation und Bewertung dieser Ausbildungsform sowie das (Über-)Prüfen entsprechender Kompetenzen. Zukünftig wird mehr Raum für die interprofessionelle Fort- und Weiterbildung nötig sein. Strukturierte Forschungsprogramme sind essenziell, um die Effekte von interprofessioneller Ausbildung im DACH-Raum nachweisen zu können.

In diesem Positionspapier regt der GMA Ausschuss für Interprofessionelle Ausbildung an, dieses Thema weiter voranzubringen und möchte gemeinsam mit anderen entstandenen Netzwerken weiterhin für eine Stärkung und Intensivierung von interprofessioneller Ausbildung und Zusammenarbeit im Gesundheitswesen sorgen.

## 1. Introduction, aims and method

In this position paper the terms interprofessional learning (IPL), which takes place in the interaction between members or students of different (health) professions, and interprofessional education (IPE), which formalizes IPL [1], are subsumed under the term IPE. IPE is meant to prepare for effective interprofessional collaborative practice (IPCP) on the part of the (health) professions – primarily in the context of an ever more complex healthcare system with demographic and technological transformations. Furthermore, the current challenges show that successful IPE and IPCP are essential requirements for high-quality healthcare. Medical students and trainees in the health professions should therefore be prepared for the challenges of an interconnected, analog and digital healthcare.

The aim of this position paper is to present the current state of IPE implementation in the German-speaking countries (Germany, Austria and Switzerland) since the 2015 position paper [2]. This present paper focuses first on the current national developments in the three countries, identifies other main areas of focus regarding IPE internationally and reflects on them. Finally, this position paper sums up important aspects and makes recommendations that should enable a sustainable implementation of interprofessional learning in the curricula of health care professions. Suggestions for advanced training and post-graduate education are likewise listed for all medial school graduates and trained healthcare professionals who work with patients and their families.

Based on the position paper in 2015 [2], the continuous work done by the working groups of the German Association for Medical Education’s (GMA) Committee on Interprofessional Education (in German: Ausschuss für interprofessionelle Ausbildung, AIA) was incorporated into this update so that the relevant topics were jointly explored and discussed with the working groups. In addition, a search of Medline in Pubmed was conducted mainly and equally by the first, second and last authors. The results were discursively supplemented and verified by the other authors. Inclusion criteria were: systematic reviews in English published between 2015 and 2021 (as of: 21/06/21) containing “interprofessional education” in the title. Narrative reviews were excluded to ensure both a high-quality evidence base and the relevance of the interprofessional trends under investigation. All of the systematic reviews underwent explorative screening to capture the most important trends in IPE/IPCP. Overall, 26 reviews [3], [4], [5], [6], [7], [8], [9], [10], [11], [12], [13], [14], [15], [16], [17], [18], [19], [20], [21], [22], [23], [24], [25], [26], [27], [28] were included. The core aspects of IPE/IPCP identified through the research are discussed in the separate segments of this position paper. The 3P model (presage, process, product), proposed by Biggs in 1993 [29] and adapted for IPE by Freeth & Reeves 2004 [30], served to structure the information as presented here. In general, when presenting and discussing the results, an attempt is always made to compare the German-speaking countries with the international data where possible using the available scientific literature.

## 2. Interprofessional education in Germany, Austria and Switzerland

### Germany

Prior to 2015, course offerings in interprofessional education entailed only a few pilot projects. In recent years in Germany there is a trend toward more IPE programs with the initiation of numerous educational projects, some anchored in the curriculum, which enable several professional groups to learn together [31]. Additionally, aspects of IPE have been included in the definitions of objectives and competencies in the regulations governing study and training in the health professions. Interprofessionality is mentioned now more frequently as a topic, for instance, in the rules governing teaching and testing in the nursing professions (*Ausbildungs- und Prüfungsverordnung für die Pflegeberufe* [https://www.gesetze-im-internet.de/pflaprv/BJNR157200018.html]), in the revised version of the National Competency-based Catalogue of Learning Objectives in Undergraduate Medicine (NKLM 2.0, [http://www.nklm.de], e.g., chapter 8), and in the draft of the new medical licensing regulations (*Approbationsordnung*) [32]. Thus, there are now for the first time mandatory guidelines on IPE in selected rules and regulations governing education of health care professionals in Germany.

Nevertheless, there is still no national concept for the implementation and realization of IPE. The long-term and longitudinal curricular anchoring of interprofessional formats thus poses a challenge for many faculties after the expiration of initial funding and external sponsorships, for example, by the Robert Bosch Stiftung [33]. Concerns exist that initiatives in the area of IPE, as well as its research, will remain without effect in the long term if the structural conditions in healthcare and educational institutions do not support sustained implementation. Similarly, IPE needs to take even more account of professional diversity, especially also independent of medical schools, which have so far led the way. Given the plurality of educational institutions (universities, universities of applied sciences, vocational schools), there is a need to actively expand initiatives to include other institutions involved in training healthcare professionals, in addition to the medical faculties.

Furthermore, there is a current lack of suitable methods to transfer interprofessional learning content to actual practice in later professional careers. There are indications that sustainable effects of IPE to improve health care practice need to be supported by appropriate measures to promote IPCP [34], [35]. Measures to create missing links between interprofessional educational work and collaborative practice as well as common taxonomies must be the focus in the future to sustainably establish IPCP in care practice in Germany.

#### Austria

In Austria, education and training in the health professions are still primarily monoprofessional in nature according to the authors' assessment, although the relevant regulations strengthen the option to allow interprofessional approaches, mainly at individual institutions or in the context of international collaborations. The importance of IPCP is pointed out at different levels as necessary to ensure integrated healthcare [36].

Studies in connection with IPE or IPCP among the health professions are still rare in Austria. Even though IPCP and interprofessional discourse are described for the individual health professions in the 2020 brochure published by the Federal Ministry for Social Affairs, Health Care and Consumer Protection [36], a more in-depth analysis of this topic appears to be absent. In a position paper by the Austrian Society for General Practice and Family Medicine, it is pointed out that interprofessional collaboration can be successful while maintaining the significance of the work reserved for a particular profession and to do this, a process-based restructuring of work processes appears necessary [37].

Also at the level of educational institutions, the greatest challenges in this context continue to be of an organizational nature: for example, the design of joint learning settings between universities and universities of applied sciences; the different processes and structures at the various educational institutions often pose a hurdle. In the meantime, there are numerous “good practices” on IPE at the universities of applied sciences, which are also curricularly anchored in bachelor and master programs [38]. 

#### Switzerland

In Switzerland, there are favorable conditions for IPE, which have been further advanced in recent years and supported in particular by political strategies as well as efforts on the part of educational institutions [39]. In addition, the future-oriented image of IPE in Switzerland is complemented by initiatives of the Swiss Academy of Medical Sciences (SAMW) and accompanying national developments (e.g., Platform for Interprofessionalism or SwissIPE) [40]. For instance, in 2014, the SAMW revised the charter on interprofessional collaboration and published it in September 2020 as Charta 2.0 on interprofessional collaboration in healthcare [41].

In 2013 the Swiss Federal Council adopted the strategy outlined in Healthcare 2020 as a response to the challenges facing the healthcare system. Under the concept of healthcare quality, this strategy underlined the education of healthcare professionals along with fostering interprofessionality as an important point. Based on the results of this funding phase, the Swiss Federal Office of Public Health has bundled the knowledge and insights from 18 research mandates and formulated recommendations for practical implementation in the form of policy briefs. Calls have been expressed to strengthen IPCP in outpatient, inpatient and psychiatric care as well as IPE in education [42]. In addition, a Health Professions Act has been initiated, and the Medical Professions Act has been revised so that both of these acts now refer, at least indirectly, to the importance of IPCP or its consideration in education. To provide information about interprofessional projects in Switzerland, the Federal Office of Public Health introduced a publicly accessible platform, which now lists 76 interprofessional projects ([http://www.bag.admin.ch/modelle-interprof], last verified on 15/06/2021).

On the level of the educational institutions, the curricular frameworks for upper-level schools (tertiary level B) and the curricula for vocational schools (secondary level II) underscore the importance of IPE competencies for the role of the “collaborator” According to the CanMEDS role model [43], these have been incorporated into the final competencies of the health professions and human medicine [44].

However, the analysis in a current position paper by the Careum foundation [40] shows that there will be ongoing needs for action in the coming years to further advance the development of IPE. In particular, the main focus is on identifying and defining interprofessional competencies, standardizing existing competency models applied at different sites, anchoring IPE in the curriculum, and including IPE as a criterion for accreditation. It also appears important to train facilitators and monitor projects with high-quality research. As part of the promotional strategy of the Swiss Federal Office of Public Health, the Competence Network Health Workforce (CNHW) [45] was established, from which a working group on interprofessional competency frameworks has emerged. The aim of both groups is to find consensus on interprofessional competencies and to strengthen IPE.

#### Summary of the status of interprofessional education in the German-speaking countries

There has been a clear growth in IPE programs and courses over the past six years in the German-speaking countries, not only as a result of political or community-based funding initiatives (Germany: Robert Bosch Stiftung – “Operation Team”; Switzerland: Federal Office for Public Health – funding program for the “Skilled Workers Initiative Plus”), but also local initiatives. In Switzerland there are national frameworks for specific areas of IPE (e.g., as a result of the passage of the act governing health professions and the revised act governing the medicinal professions), and in Germany IPE can be found in the rules and regulations governing training and education in individual health professions, making the political impetus behind more IPE visible. In Austria, it is evident that the importance of the topic is being taken up – but the implementation of IPE in the framework conditions of education is still largely pending. All of the German-speaking countries share in common that many IPE initiatives must be communicated in the wider healthcare context, and these initiatives should be firmly anchored.

A comprehensive overview of further developments in the German-speaking countries is found in Ewers & Walkenhorst [46].

## 3. Specific core aspects of interprofessional education

**Figure 1:**
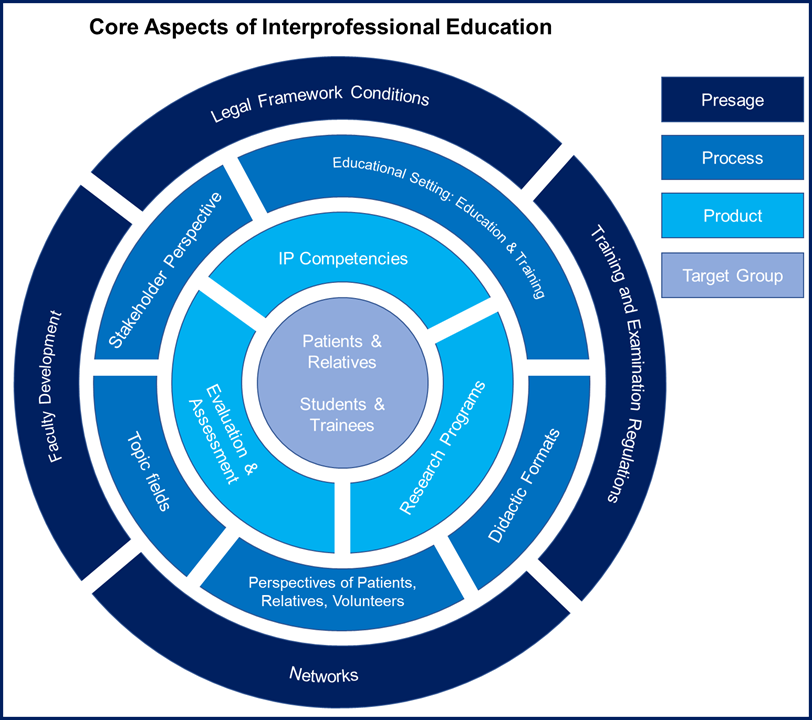
Core aspects of interprofessional education according to the 3P model [29], [30]. (Authors’ illustration)

In the following, the essential core aspects for the future focus of IPE in the German-speaking countries are classified based on the 3P model by Freeth & Reeves [30] (see figure 1 (Fig. 1)). The focus of every IPE program is on the trainees/students and patients with their family members as the target group.

### Presage – general frameworks for interprofessional education

#### Legal frameworks

As mentioned in the country-specific sections, there has been success in strengthening interprofessionality in education through amended or newly drafted legislation on education and testing, lists of learning objectives, criteria for accreditation, and statutory provisions in the German-speaking countries. However, a lack of funding, e.g., for human resources, makes implementation that much more difficult at the educational institutions.

Broadening the view beyond the German-speaking region in the relevant literature, a review [15] of 16 articles derives several recommendations for action (e.g., embedding IPE in specific learning environments, starting IPE early during training, structured and longitudinal evaluation of interprofessional competencies) for the further development of IPE, first from a global perspective and then specified for the Asian region. In another review with an international perspective [7], it is evident, based on a total of 65 studies, that differently developed countries demonstrated different capacities to advance IPE more quickly and successfully. However, at present these initiatives generally appear insufficient to achieve the global health goal of establishing IPCP worldwide.

##### Networks

Various organizations and networks have emerged in the German-speaking countries in recent years pursuing the goal of promoting interprofessionality from the micro level (implementation of local interprofessional teaching projects) to the macro level (anchoring IPE in legal regulations and widespread implementation). The harmonization of these efforts by different initiatives and a joint influence on political support for IPE form the central tasks of future networking. The AIA of the GMA is eager to jointly discuss the developments and challenges of IPE with existing networks and ascertain recommended measures and concrete actions.

Notable initiatives include, for instance, “IP Health” (Society for interprofessional healthcare, “Gesellschaft für IP Gesundheitsversorgung e. V.”), another network spanning Germany, Austria and Switzerland, the “Expert Commission on Interprofessionality” of the higher education network for health professions (“Hochschulverbund für Gesundheitsberufe e. V.”) in Germany [47], and, at the institutional level, the “Network for Interprofessional Education” at the Charité Hospital in Berlin, the “Working Group on Collaboration in Healthcare” (AG ZiGeV) in the German Network for Healthcare Research, as well as “SwissIPE” and the “Platform for Interprofessionality” in Switzerland. To be mentioned also are the “Network of Health Interprofessional” – formed as part of the projects funded by the German Federal Ministry for Education and Research (BMBF) including “Health Care Professional” at the Alice Salomon University of Applied Sciences Berlin – and “Competency Development in Healthcare workers in the Context of Lifelong Learning” (KeGL) at the University of Osnabrück. Another example of successful networking is the post-graduate research program “Interprofessional Education in the Health Professions” (ILEGRA, [http://www.ilegra.uni-osnabrueck.de]), which will be covered in more detail in the section on research.

##### Faculty development

Supportive framework conditions for IPE and IPCP must be in place not only at the organizational-structural level, but also at the faculty level. In order to set up sustainable IPE programs, faculty development with the support of all stakeholders is necessary. Executive leadership should have an interprofessional composition and create flexible structures that support and facilitate the implementation of IPE projects. Time, human resources, and attractive incentives (e.g., “Clinical Teacher” as a career option) must be made available in order to anchor IPE permanently in the curricula of the healthcare professions [48].

Educators in the area of interprofessional education should be specially trained for their work, particularly regarding interprofessional competencies. They should understand and respect professional differences and be able to lead discussions and show respect for the individual contributions of each profession. Interprofessional training wards have led to the first suggestions for standardizing basic training for interprofessional mentors (often referred to as facilitators) [49], [50], which also allows transfer of this concept to other interprofessional teaching strategies. All health professions educators should understand the importance of interprofessional team-based care and discourage an exclusively monoprofessional care logic [48]. As emphasized in the 2015 position paper, this continues to appear one of the greatest challenges facing IPE, even if the new legislation gives rise to expect pressure to act. It is therefore essential to have supportive frameworks for all stakeholders, including the educators in order to create and maintain IPE programs [2].

Cooperation with healthcare institutions should be expanded. When creating comprehensive interprofessional clinical learning environments, IPE goals should be divided between the people responsible for education and those for the clinical area in order to support the transfer of IPE to clinical practice [48].

#### Process – procedural aspects in the area of interprofessional education

##### Topic fields

In the international context, IPE is examined in reviews on the management of diabetes [13], [26], [28], mental health [20] and for dementia [4]. One review focuses explicitly on IPE in the primary care setting [3]. Similar topics are also mentioned in the recommendations made by the Institute for Medical and Pharmaceutical Examination Questions (Institut für medizinische und pharmazeutische Prüfungsfragen (IMPP)) and the Robert Bosch Foundation [51] regarding the design of interprofessional education at German medical schools. Likewise, in Switzerland, as part of the final reports by the funding program “Interprofessionality in Healthcare”, various topics are considered suitable for interprofessional education: it is stated that topics affecting all professional groups could be used as learning content, for example, health competence, digitalization, ethics, communication, or the inclusion of the patients ([45], p.7). Basically, all topics that require collaboration and cooperative problem solving in healthcare are suitable for IPE.

##### Educational setting

Internationally, reviews have examined undergraduate education as well as continuing education in both clinical and theoretical settings. A steady increase is seen in the focus on interprofessional advanced training and post-graduate, continuing education and on improving collaboration between healthcare workers. It is precisely these advanced training and post-graduate teaching strategies [51], which are situated directly in the workplace, that are purposeful for ensuring comprehensive patient care in the future. Although local initiatives already exist in the German-speaking countries [52], [53], [54], there is still an absence of more elaborate strategies for individual aspects of the healthcare system, such as is the case for a region in New Zealand. Here the implementation of specially trained interprofessional teams was able to prevent hospital stays through efficient, outpatient, interprofessional care [55].

##### Perspectives of the stakeholders and professional groups

In international reviews, other professions that are gaining more importance in IPE are the dental [14], [26], social [5], [6] radiooncological [24] and pharmaceutical [9] professions, alongside the medical, nursing and therapeutic professions. Systematic reviews of other professional groups were not found. Reviews were undertaken for medical [25], nursing [16] and pharmaceutical education [9] that looked at the image of IPE in each profession-specific context.

Ulrich and Breitbach [56] and Ulrich and Kaap-Fröhlich [57] encourage the inclusion of even more professional groups and disciplines, such as sport scientists and biomedical lab diagnosticians, to further optimize patient care. In the committee’s view, this applies to other professions and disciplines, such as health science, and has already taken place at individual sites [58]. Nevertheless, cooperation between professions beyond medicine, nursing and physiotherapy in the context of interprofessional teams in the German-speaking countries appears to be under-developed overall. Professional groups such as those in medical technology, medical therapy, and social work could also be included in a more sustained and substantial manner. Moreover, experts who serve as decision-makers in the healthcare system but who are not directly involved in providing care, such as health economists or those active in politics or the insurance industry, could also be included so that they can each contribute their expertise.

##### Perspectives of patients, volunteers and family members

One review focused on how patients benefit from IPE [13]. In the German-speaking countries patients at the moment have a more subordinate role in interprofessional education. Efforts to include patients in the planning and execution of interprofessional education are very rarely described in the literature. Approaches in Great Britain [59] that include patients could offer examples for a more active role for patients in the teaching and training of the health professions. Patient participation is accommodated at different stages and can even encompass inclusion in institutional decisions regarding education, evaluation and curriculum development for the health professions. Given the goal of improved patient care through IPE, the experience of patients when receiving care should be systematically taken into consideration by the different professions and possibilities for equal participation in IPE on the part of patients should be developed further. The Careum Summer School [60] in Switzerland shows a possible approach to extracurricular interprofessional teaching with patients and their relatives. In Switzerland there are also projects that study and investigate the relatives of patients and care volunteers as members of the interprofessional team [61].

##### Didactic formats

Two trends on the didactic level can be recognized in the literature:

1. online-supported formats, and
2. practical training settings, such as training wards. Internationally, online teaching is being investigated in terms of IPE: the use of social media [21], online learning facilitation [19], use of web-based training [8] or the influence of a learning environment (e.g., Team STEPPS) on IPE [17].

Kuhn et al. recommended in 2018 that the digitalization of education in the health professions should be addressed in an “interprofessional” manner [62] and made appropriate recommendations. To implement these recommendations, the interprofessional Digital Health & Education Multiplicator program was established in 2020 [63]. The aim is to train multiplicators inside healthcare institutions who then facilitate change and transformation programs using interactive digital teaching and learning formats.

##### Interprofessional training wards

The first interprofessional clinical training wards were implemented in Freiburg, Heidelberg and Mannheim in 2017 [64], [65], [66] and in Zurich in 2018 [67]. Other training wards now exist in Bonn, Bremen, Hamburg, Munich, Nuremberg, Berlin and Marburg or are being planned, and this is also the case in Switzerland. Funding for interprofessional training wards is provided by third party funding as well as through budget financing.

An important aspect of interprofessional training wards is the preparation for starting a career in an interprofessional team. Studies show that the participants learn not only interprofessionally but also professionally [68] and that patients and their family members perceive the care on the training wards to be excellent [69]. The participants perceive their deployment on such training wards very positively [70]. On the national level in Germany, the importance of interprofessional training wards is being emphasized by the national medical students’ body [https://www.bvmd.de/portfolio-items/interprofessionalitaet/?portfolioCats=110] – Bundesvertretung der Medizinstudierenden – and the IMPP [71]. The concept of the interprofessional training ward as an educational model is also being recommended in Switzerland [67]. Challenging are the permanent curricular implementation of the new training wards, especially after the initial funding has ended, and the expansion of the program so that as many medical students and healthcare trainees as possible can take advantage of the opportunity to experience this educational format. Possibly, established monoprofessional teaching and learning formats, such as clinical internships or mentorships, can be supplemented with interprofessional elements in order to enable cooperative learning for as many students and trainees as possible [72].

##### Excursus: COVID-19 pandemic and interprofessional education

As was the case with monoprofessional education, interprofessional learning has been heavily curtailed since early 2020 by the challenges of the COVID-19 pandemic, with many interprofessional courses being reduced, not offered at all, or offered in altered forms [73]. Caring for severely infected COVID-19 patients highlighted the need for interprofessional collaboration, a need that was mostly well met even in the challenging situation and opened up new perspectives on cooperation [74]. In the committee’s view, this unusual situation especially emphasizes the necessity to implement strategies for interprofessional education. These strategies provide preparation for intense collaboration of the health professions and the optimal inclusion of the expertise of all participants. Overall, the situation during the pandemic demonstrates, once more, the importance of widespread implementation of IPE, which is, in part, being pursued as called for in the legislation governing education and training. To date there have been few publications that examine the context of interprofessional care during the COVID-19 pandemic. In the special issues published by the GMS Journal for Medical Education on teaching during the COVID-19 pandemic in 2020/2021, only five of 40 publications in the first issue and two of 31 publications in the second issue take up the topics of interprofessional target groups or interprofessionality. This shows that some initiatives can be found to address the pandemic and allow for innovative interprofessional education approaches under pandemic conditions, which are likely even mandatory for the safe care of COVID-19 ill individuals. More programs and courses are urgently needed that go beyond undergraduate education and focus on advanced training and post-graduate education in order to anchor IPCP in daily clinical practice during the COVID-19 pandemic. There are also a few international examples regarding this [75], [76], [77], but no nationally defined strategies, probably due to the challenges of the pandemic.

#### Product – products, results and research on interprofessional education

##### Interprofessional competencies and interprofessional identity formation

In recent years, there has been a focus on competency-based teaching in monoprofessional education [44], [78]. Such approaches to IPE do also exist. While many frameworks on IPE already exist internationally, primarily in the English-speaking countries [79], [80], [81], there is no comprehensive framework in German yet. Definitions and descriptions of competencies have been developed in Switzerland since 2017 based on analyses of existing frameworks and expert surveys that could serve as a starting point for a German framework [82]. In Germany the interprofessional learning objectives contained in the National Competency-based Catalogue for Undergraduate Medical Education (NKLM 2.0) offer a basis for competency-based curriculum development for the study of medicine at the university level. However, interprofessional competencies and learning objectives are still unequally represented in the legislation governing the professions and/or the licensing regulations for the health professions (see above). Uniform and consistent definitions and wording are worth striving for to enable IPE on the level of the competency descriptions. This can be done in a consensus-building process in which cross-professional competencies are defined by representatives of the different professions, including students and trainees (and also within institutions, see e.g. [83]).

The implementation of interprofessional competencies should definitely be part of the mandatory curriculum of all professions involved and should not be limited to elective courses. A systematic review of the nationally defined competencies in terms of curricular mapping facilitates the accreditation and certification of interprofessional competency acquisition.

The training of teachers and multiplicators should also be based on previously defined interprofessional core competencies [48]. Competency-based and practical formative and summative assessment formats, such as the Mini-CEX or OSCE, should constantly be taken into consideration from the start.

In addition, interprofessional competencies promote and foster interprofessional identity formation [84] which, in turn, supports interprofessional socialization for multiprofessional collaboration in clinical practice.

##### Evaluation, assessment and evidence

In view of evaluations, the translation of existing validated English interprofessional survey instruments (e.g., UWE-IP, ISVS) [85], [86] into German, and in some cases their validation, is good news. This enables comparisons of the effects [85] of similar courses and programs. It is challenging, however, to adapt these survey instruments to local conditions in order to evaluate as many aspects of a specific interprofessional setting as possible. In general, the quality of IPE cannot be solely represented by one survey or a one-dimensional evaluation because it involves a complex multilayered construct. A demand for harmonization of accompanying research is therefore essential. With the creation of many new interprofessional initiatives there is now the opportunity to evaluate these nationally or even internationally and, based on the comparative results, to identify best-practice recommendations for the future orientation of IPE in the German-speaking countries and to map the development of an interprofessional identity [87].

This issue is also addressed from an international point of view, whereby different evaluation tools are scrutinized in terms of their quality and decision-making aids are given for selecting a suitable survey instrument depending on the focus of the evaluation [27].

Most of the international reviews identified address overarching aspects of the evidence or evaluation of IPE. The evidence base continues to be challenging, but has been improved in recent years: The reviews by Reeves et al. (2016), Visser et al. (2017) and Spaulding et al. (2019) along with the meta-analysis by Wang et al. (2019) include an overall rather large number of high-quality studies, with 46, 65, 19 and 16, in their analyses [6], [10], [22], [23]. In particular, the evidence shows positive effects of IPE with regard to interprofessional attitudes and perceptions [6], [23] as well as knowledge and skills [6], and in a more recent review also in regard to changes in individual collaborative behavior [22]. Moreover, there are indications that successful IPCP increases work satisfaction and possibly the satisfaction of patients and improves the care they receive [88], [89]. There is less evidence and a need to catch up in the research on IPE effects regarding collaborative changes in the particular institutions, on the effects on patients, and on the sustained effects of IPE [6], [23].

##### Research programs

An example of successful development and fostering of a new generation of academic researchers is ILEGRA, a graduate program that is run in cooperation between the University of Osnabrück and the LMU Hospital (Ludwig Maximilian University in Munich) and funded by the Robert Bosch Stiftung. Since October 2018, 16 fellows from different health professions have been working on dissertation topics related to teaching, examination, and evaluation in the context of IPE or in health care practice. ILEGRA is the first graduate program nationally and internationally to focus on interprofessional education in the health professions.

In 2016 the Swiss Federal Council resolved to set up the nationally funded program “Interprofessionality in Healthcare” [90]. The focus of this program, that run from 2017 to 2020, was firstly on research programs that would create knowledge bases to promote IPCP in particular. A second main point was the documentation of good practice models which could serve as examples – which can be assessed online at the website of the Federal Office of Public Health – and could provide incentives and ideas for developing new interprofessional courses or programs. “Interprofessionality in Healthcare” was finished in the end of 2020 after the most significant results and recommendations were compiled in the form of policy briefs for the areas of outpatient care, clinical care, the interface between psychology and somatic medicine, and for the field of education [42].

In summary, there is also continuing interest in and development of IPE worldwide in different settings, for different professional groups and regions. Alongside Canada and the US, the Scandinavian countries continue to lead in terms of IPE, in particular Sweden and Denmark [91]. In other places it is individual institutions [92] or, at least, points of orientation such as the CanMEDS model [43] that provide examples for IPE. Established national or international curricula are virtually nonexistent despite the emphasis placed by the WHO [93] on the importance of IPE more than ten years ago. Determining whether there are political, cultural or purely practical obstacles here and how these can be overcome must be the focus of the coming years.

## 4. Limitations

Although IPE is viewed as a requirement for successful later interprofessional collaboration, data is missing on long-term effects of mostly unique interprofessional teaching and learning interventions. A longitudinal approach to IPE within a particular institution (e.g., university) and coordinating individual teachers/courses can be helpful in terms of this. As is the case in monoprofessional education, IPE faces the challenge of competency-based teaching and assessment, perhaps even more so because many of the communication skills are difficult to teach and assess in a standardized manner. If possible, cross-faculty examination formats should be used here to enable comparability. Costs for implementation are incurred through planning and interprofessional staffing. Many faculties are unable or unwilling to provide these resources, especially since little data exists on the cost-effectiveness of interprofessional training programs. A national network, proof of competency gains, and, ideally, improved patient outcomes would be helpful in this sense, also to enable further funding. Overall, the topic of interprofessionality is taking an ever more important role in the German-speaking countries - now the time to set the future course for interprofessional, patient-centered education in medicine and the health professions.

## 5. Recommendations

The committee recommends the following in reference to *P1* “*presage*”:

1. Consider existing and new interprofessional teaching and learning projects in the budgets of educational and healthcare institutions.
2. Strengthen networks dedicated to further developing interprofessional education and the interactions and affiliations among them.
3. Create basic conditions that enable new forms of education and cooperation in education and healthcare.
4. Recognize and support the necessity of IPE and IPCP to meet future challenges and in future pandemics, as is now made evident by the COVID-19 pandemic.
5. Define the legal frameworks for IPE, e.g., in the rules and regulations governing education, profession-specific legislation, etc. and to anchor IPE there.
6. Organize and establish educational programs on interprofessional topics for teachers and facilitators.
7. Compile national/international standards for educational programs on interprofessional topics for teachers and facilitators.

The committee recommends in reference to *P2* “*process*”:

1. Advance an expansion of interprofessional training wards for all students and trainees in the health professions.
2. Strengthen new teaching and learning formats, for instance, online courses.
3. (Include more vocational training and university degree programs in IPE.
4. (Give patients, their family members and volunteers an active role in IPE.
5. Make interprofessional courses and programs part of advanced training and post-graduate education, e.g., in medical specialization or training to become an advanced practitioner.

The committee recommends in reference to *P3 produc*t:

1. Support the anchoring of interprofessionality not only in the rules and regulations governing educational degree and training programs, but also in practice.
2. Develop a German, cross-professional framework on interprofessional competencies.
3. Give competency-based assessment formats an interprofessional focus and implement them to the extent possible for all the professional groups involved.
4. Support and conduct nationally and internationally coordinated research on the effects of interprofessional education.

## 6. Conclusion and outlook

In the seven years since publication of the first position paper on interprofessional education by the AIA of the GMA, an impetus arose to realize various interprofessional teaching and learning strategies. There have also been first political impulses to anchor IPE in core curricula and the regulations governing university education and vocational training.

In the future it will be essential to not just offer unique, locally concentrated courses in the sense of teaching and learning projects, but also to overcome the project status of interprofessional training nationally and internationally and to transfer it to the phase of secure permanence and funding. The initial political mandates must therefore be implemented in a broad sense of interprofessional education and, likewise, in advanced training and post-graduate programs. Including additional professional groups in interprofessional education can only be positive. Further research, especially on the sustainability and transferability of IPE to IPCP nationally and internationally, is necessary. This assumes that research on interprofessional education and advanced training will be anchored in the grant programs of third-party funders.

## Notes

The position paper was acepted by the GMA executive board at 28-02-2022.

Sylvia Kaap-Fröhlich, Gert Ulrich and Sebastian Bode contributed equally to the manuscript.

## Competing interests

The authors declare that they have no competing interests.
